# Evaluation of the service quality and diagnostic performance of syphilis self-test kits purchased from E-commerce platforms in China

**DOI:** 10.1038/s41598-026-44567-5

**Published:** 2026-03-22

**Authors:** Fangzhi Du, Hongchun Wang, Yue Xi, Xiaoyu Zhu, Kai Chen, Ruili Zhang, Xu Zhang, Xiaoli Zheng, Shaochun Chen, Weiming Tang, Qianqiu Wang

**Affiliations:** 1 Hospital for Skin Diseases, Institute of Dermatology, Chinese Academy of Medical Sciences and Peking Union Medical College, Nanjing, China; 2 Department of Dermatology, The Second Affiliated Hospital of Nanjing Medical University, Nanjing, China; 3 University of North Carolina Project-China, Guangzhou, China

**Keywords:** Syphilis, Self-testing, Quality scale, Diagnostic performance, Diseases, Health care, Medical research

## Abstract

Syphilis self-testing (SST) facilitates screening accessibility among key populations (such as men who have sex with men, female sex workers, etc.). SST kits are readily available on several prominent e-commerce platforms in China. This study aims to compare the differences of diagnostic performance between kits obtained online or based on facilities, which provided evidence for the expanding application of SST in China. Utilizing the HONcode standard, we developed a quality assessment scale to evaluate the service quality of online SST kits. Furthermore, we assessed the diagnostic performance of them compared with facility-based kits, using TPPA as the gold standard. The online sales data showed that the after-sales reviews for SST kits accounted for only 5.5% of HIV self-testing kits and 17% of HIV/syphilis dual-testing kits. Quality assessment results indicated scale scores ranging from 31 to 44 for these SST kits, reflecting satisfactory service quality. The sensitivity and specificity of eight online kits were 94.85%-100% and 98.90%-100%, respectively, and the nine facility-based testing kits were 91.91%-100% and 97.81%-100%, respectively. The online SST kits demonstrated satisfactory quality and diagnostic performance, which provide evidence for promoting the utilization among key populations in China.

**Supplementary Information:**

The online version contains supplementary material available at 10.1038/s41598-026-44567-5.

## Introduction

According to the latest report from WHO, syphilis had an estimated 7.1 million newly infected cases per annum in 2020^1^, being one of the most common sexually transmitted diseases (STDs) globally. In China, there were reported over 480,000 new cases of syphilis in 2021. The implementation of the National Syphilis Prevention and Control Plan (2010–2020) significantly enhanced population-wide screening coverage, resulting in an average annual decline of about 10.39% among primary and secondary syphilis in the past ten years^2^.

However, recent researches reveal persistently high syphilis prevalence among high-risk populations in China, including men who have sex with men (MSM) and female sex workers (FSWs), with reported prevalence rates of 12.0% and 5.2%, respectively^3,4^. According to the recommendations of the Chinese Center for STD Prevention and Control, such populations should be tested for syphilis every 3–6 months^5^. However, due to privacy protection, social discrimination and stigma, previous negative health-care experiences, reluctance to disclose sexual practices, these populations have limited access to health-care facilities, and are at higher risk of losing follow-up and lower uptake of testing for syphilis as mandated^6–8^. In low- and middle-income countries, the proportion of MSM undergoing syphilis testing remains significantly low. Research indicates that merely 30% of MSM in China have ever undergone syphilis testing^9,10^. In addition, it is necessary to provide regular syphilis screening in sexually active people, but routine on-site testing is not easy to accept. Therefore, syphilis self-testing (SST) is an important choice according to the recommendation of the WHO.

Self-testing may provide an easier accessible healthcare pathway to subgroups discriminated against or less reachable by the healthcare system, including MSM and FSW, and reduce cost and improve adherence to therapeutic and preventive regimens^11,12^. WHO has recommended self-testing for HIV since 2016, and has since expanded self-testing to new disease areas^13^. Recent studies have also shown that about 25% of MSM and 6% of FSW who had ever received syphilis testing had conducted SST in China, indicating its significant acceptance and promising implementation potential^14,15^. Currently, Chinese SST kits are predominantly distributed through e-commerce platforms. Purchasing these kits online eliminates the in-person interactions and enhances privacy protection. There are many manufacturers of these products with different prices and diagnostic performance. Crucially, there exists a paucity of systematic evaluations regarding product reliability, operational standardization, and diagnostic accuracy. These unaddressed quality control issues pose substantial barriers to the broader implementation and scale-up of SST initiatives in China.

Therefore, we intend to evaluate the service quality and diagnostic performance of major SST kits sold on e-commerce platforms, to promote the application of SST in some important populations, improve the coverage of medical services, and effectively control syphilis.

## Results

### Information on 8 online SST kits

We selected 8 brands of SST kits with the highest after-sales evaluation from e-commerce platform ‘JD.com’, which were Accu News, Wondfo, WELLDAY, Abbott, JISSBON, LOVCAE, AIJI, and KANGHUA. The price of these 8 SST kits ranges from 16.47 (2.34$) to 37.05 (5.26$) RMB. The testing package includes a testing tool and a blood sampling tool (disposable end blood sampling needle, disposable disinfection cotton pad, Band-aid). Among these 8 kits, the combined packaging of the testing kit and blood collection tool is used in Accu News and AIJI. All kits are suitable for testing whole blood, serum, and plasma from the fingertips. However, the test results may be affected among testers with high cholesterol, high lipids, high hemoglobin, high heme, etc. Each kit has a different impact threshold for these indicators. Seven of these kits describe situations where no cross-reaction occurs. All kits provide instructions for the test process and interpretation of test results. However, only Accu News and Abbott provide instructions for the use of blood sampling. Five of these kits describe a 100% positive and negative reference conformity rate, and three kits describe over 99.5% total clinical compliance rate. The details of these 8 self-testing kits are shown in Sup Table 1.

### Service quality scores of 8 online SST kits

As shown in Table 1, the Service Quality scores of eight online SST brands were 35.5 (IQR: 33.5–40.5). Although these SST kits fail to meet all criteria, they demonstrated good performance in median quality scoring. Scoring differences primarily depended on the presence of a blood sampling procedure instruction, the inclusion of a notice stating that positive results do not confirm clinical diagnosis and negative results do not rule out syphilis infection within 3 months in high-risk populations, and the availability of professional clinical consultation for after-sales service.

**Table 1:** Scores scale of eight online SST kits.

Provider					Eight online brands of SST kits in JD.com							
Indicator	Explanation	Answer	Rubric	Clinical Weight	Accu News	Wondfo	WELLDAY	Abbott	JISSBON	LOVCAE	AIJI	KANGHUA
Pre-Testing												
Brand	Brand popularity (‘unknow’ is defined as brand with less than 10,000 after-sales reviews)	International labels	1	**3**	1.5	1.5	1.5	3	1.5	1.5	1.5	1.5
		Well-known in China	0.5									
		Unknown	0									
Price	Price level (‘low’ is defined as price lower than facility-based testing (30 yuan))	Low	1	**2**	2	0	2	0	2	2	2	2
		High	0									
Delivery	The speed of delivery (‘fast’ is defined as delivery within 2 days)	Fast	1	**2**	2	2	2	2	2	2	2	2
		Slow	0									
Packaging	Whether to protect privacy (do not show any package information)	Yes	1	**2**	2	2	2	2	2	2	2	2
		No	0									
	Are all test contents packaged integrated?	Yes	1	**2**	2	0	0	2	0	0	2	0
		No	0									
Testing												
Qualification	Is the test offered the National Medical Products Administration (NMPA) approved?	Yes	1	**5**	5	5	5	5	5	5	5	5
		No	0									
Test Type	Does the test type offered reflect those recommended by the Chinese STI management guidelines (NAAT, etc.)?	Yes	1	**4**	4	4	4	4	4	4	4	4
		No	0									
Usability												
Instructions	Has the type of sample required for the test been indicated (e.g. fingertip, earlobe, venous blood.)	Yes	1	**2**	2	2	2	2	2	2	2	2
		No	0									
	Whether to indicate the storage conditions of the testing kits?	Yes	1	**2**	2	2	2	2	2	2	2	2
		No	0									
	Are instructions provided for the testing process?	Yes	1	**5**	2	2	2	2	2	2	2	2
		No	0									
	Are instructions provided for the sampling process, such as fingertip blood collection?	Yes	1	**3**	3	0	0	3	0	0	0	0
		No	0									
	Whether the interpretation of testing results is provided?	Yes	1	**3**	2	2	2	2	2	2	2	2
		No	0									
	Whether to indicate any special circumstances that might have influenced the results (e.g. bilirubin, cholesterol, hemoglobin and other indicators may affect the outcome judgment ) or cross-reaction?	Yes	1	**2**	2	2	2	2	2	2	2	2
		No	0									
	Whether to indicate the limitations of the test, such as the test can only be qualitative but not quantitative detection; If positive, go to the hospital for further examination; Negative does not completely rule out the possibility of infection.	Yes	1	**2**	2	2	2	2	2	2	2	2
		No	0									
	Whether to provide product performance indicators, including negative and positive reference conformity rate, clinical positive conformity rate	Yes	1	**2**	2	0	0	2	2	2	0	2
		No	0									
Follow-up												
Result Interpretation	Are the results interpretation easy?	Yes	1	**3**	3	3	3	3	3	3	3	3
		Somewhat	0.5									
		No	0									
Support Contact	Is there an option to contact support for further questions?	professional clinicians	1	**4**	2	2	2	4	2	2	2	2
		customer service	0.5									
		NO	0									
Futher medical services	Sexual health counseling services, referral recommendations, sexual partner notification recommendations	Yes	1	**2**	0	0	0	2	0	0	0	0
		No	0									
Total Quality Score		Max Score = 50			40.5	31.5	33.5	44	35.5	35.5	35.5	35.5

### After-sales evaluation of 8 online SST kits

As shown in Sup Table 2, the number of total after-sales comments for all products from these eight brands ranged from 10,000 to 1 million. Among them, the number of after-sales comments of syphilis testing kits was 14,059 (ranging from 0 to 9,600), and the proportion accounted for total product comments was 1.01% (ranging from 0 to 3.52%). We assume that consumers who buy each product have the same probability of after-sales comments, and we estimate that the sales of SST kits are approximately 5.5% (0.93–21.86%) of that of HIV and 17% (1.28%-146.92%) of that of the HIV & syphilis double test kit. The most positive comments focused on the description of user-friendly operational procedures, unambiguous result interpretation, efficient logistics delivery, satisfactory customer service interactions, and effective privacy protection.

Among eight SST kits, there were a total of 142 negative comments (0–81). 50 comments were left blank. Twenty-two comments showed distrust of the test results. Fifteen comments showed that the self-testing results were inconsistent with test performed in hospital. Thirteen comments reported difficulties in blood sampling. Thirteen comments indicated that no blood sampling tool was received. Twelve comments described the poor quality of the product, including invalid test results, damaged blood collection needles, and no sample diluent, etc. Eleven comments described that the packages were damaged upon delivery, and the packaging did not protect privacy enough.

### Evaluation of the diagnostic performance of 17 syphilis rapid test kits (colloidal gold method)

As the results shown in Table 2, taking TPPA test results as the gold standard, the sensitivity, specificity, positive predictive value (PPV), negative predictive value (NPV), total coincidence rate (TCR), and kappa coefficient of 8 online kits were 94.85%-100%, 98.9%-100%, 98.54%-100%, 96.32%-100%, 97.81%-100%, and 0.96-1.00, respectively. And among the 9 facility-based kits, the sensitivity, specificity, PPV, NPV, TCR, and kappa coefficient were 91.91%-100%, 97.81%-100%, 97.14%-100%, 94.33%-100%, 96.55%-100%, and 0.93–0.99, respectively. Comparing the diagnostic performance between online and facility-based kits, we observed that the sensitivity and NPV of online ‘Abbott’ kit were significantly lower than those of facility-based kits of HIGHTOP, Biotest-syphilis, InTec, WANTAI, ABON and Biotest-Multiple test (all *P* < 0.05). The sensitivity, NPV and TCR of facility-based ‘Abbott-HIV/syphilis’ kit was significantly lower than those of online Wondfo, KANGHUA, JISSBON, LOVCAE, Accu News and AIJI (only sensitivity and NPV) (all *P* < 0.05, Sup Table 3). There was no difference in diagnostic performance among other online and facility-based kits.

**Table 2:** Diagnostic performance of eight online kits and nine facility-based kits by using TPPA as gold standarded.

Brands	Source	Sensitivity, % (95% CI)	Specificity, % (95% CI)	PPV, % (95% CI)	NPV, % (95% CI)	TCR, % (95% CI)	Kappa coefficient (95% CI)
Wondfo	Online	100.00 (97.32–100.00)	99.45 (96.99–99.99)	99.27 (96.00-99.98)	100.00 (97.99–100.00)	99.69 (98.27–99.99)	0.99 (0.98-1.00)
KANGHUA	Online	100.00 (97.32–100.00)	99.45 (96.99–99.99)	99.27 (96.00-99.98)	100.00 (97.99–100.00)	99.69 (98.27–99.99)	0.99 (0.98-1.00)
JISSBON	Online	100.00 (97.30–100.00)	99.45 (96.98–99.99)	99.26 (95.97–99.98)	100.00 (97.98–100.00)	99.69 (98.26–99.99)	0.99 (0.98-1.00)
LOVCAE	Online	100.00 (97.30–100.00)	100.00 (97.98–100.00)	100.00 (97.30–100.00)	100.00 (97.98–100.00)	100.00 (98.84–100.00)	1.00
Accu News	Online	100.00 (97.30–100.00)	98.90 (96.09–99.87)	98.54 (94.83–99.82)	100.00 (97.97–100.00)	99.37 (97.74–99.92)	0.99 (0.97-1.00)
WELLDAY	Online	97.04 (92.59–99.19)	100.00 (97.99–100.00)	100.00 (97.22–100.00)	97.85 (94.59–99.41)	98.74 (96.80-99.66)	0.97 (0.94–0.99)
AIJI	Online	98.52 (94.75–99.82)	99.45 (96.98–99.99)	99.25 (95.91–99.98)	98.91 (96.11–99.87)	99.05 (97.26–99.80)	0.98 (0.96-1.00)
Abbott-syphilis	Online	94.85 (89.68–97.91)	100.00 (98.00-100.00)	100.00 (97.18–100.00)	96.32 (92.56–98.51)	97.81 (95.53–99.11)	0.96 (0.92–0.99)
HIGHTOP	Facility-based	100.00 (97.32–100.00)	98.91 (96.11–99.87)	98.55 (94.86–99.82)	100.00 (97.98–100.00)	99.37 (97.75–99.92)	0.99 (0.97-1.00)
Biotest-Multiple test	Facility-based	100.00 (97.32–100.00)	99.45 (96.99–99.99)	99.27 (96.00-99.98)	100.00 (97.99–100.00)	99.69 (98.27–99.99)	0.99 (0.98-1.00)
InTec	Facility-based	100.00 (97.32–100.00)	98.91 (96.11–99.87)	98.55 (94.86–99.82)	100.00 (97.98–100.00)	99.37 (97.75–99.92)	0.99 (0.97-1.00)
Abbott-HIV/syphilis	Facility-based	91.91 (85.99–95.89)	100.00 (98.00-100.00)	100.00 (97.09–100.00)	94.33 (90.08–97.14)	96.55 (93.91–98.27)	0.93 (0.88–0.97)
Egens	Facility-based	98.53 (94.79–99.82)	100.00 (98.00-100.00)	100.00 (97.28–100.00)	98.92 (96.15–99.87)	99.37 (97.75–99.92)	0.99 (0.97-1.00)
WANTAI	Facility-based	100.00 (97.32–100.00)	97.81 (94.50–99.40)	97.14 (92.85–99.22)	100.00 (97.96–100.00)	98.75 (96.82–99.66)	0.97 (0.94–0.99)
hemtrue	Facility-based	99.26 (95.97–99.98)	99.45 (96.99–99.99)	99.26 (95.97–99.98)	99.45 (96.99–99.99)	99.37 (97.75–99.92)	0.99 (0.97-1.00)
ABON	Facility-based	100.00 (97.32–100.00)	99.45 (96.99–99.99)	99.27 (96.00-99.98)	100.00 (97.99–100.00)	99.69 (98.27–99.99)	0.99 (0.98-1.00)
Biotest-syphilis	Facility-based	100.00 (97.32–100.00)	99.45 (96.99–99.99)	99.27 (96.00-99.98)	100.00 (97.99–100.00)	99.69 (98.27–99.99)	0.99 (0.98-1.00)

Note: PPV, Positive predictive value; NPV, Negative predictive value; TCR, Total coincidence rate.

## Discussion

This study extended the existing literature by evaluating the performance of online brands of SST kits that are available, assessing the quality of services and the differences in diagnostic performance between online and facility-based kits. We discovered that online SST kits offer several advantages, including competitive pricing, prompt delivery, straightforward testing procedures, clear result interpretation, and good service quality assessments. Further more, all eight online test kits have excellent diagnostic performance. The sensitivity, specificity and kappa coefficient of all of them were exceeded 94.5%, 98.5%, and 0.95, respectively. The diagnostic efficacy of all evaluation kits meets the WHO’s requirements on the optimal performance of Point-of-care tests (POCT) self-testing.

Currently, the dissemination of SST kits in China is primarily through e-commerce platforms, community-based organizations, and pharmacies. In the United States, Australia, the Netherlands, and the United Kingdom, e-commerce platforms and pharmacies are the main way of obtaining SST^16–19^. However, in many low- and middle-income countries, community-based organizations and public health programs are the primary channels for obtaining SST, such as in Zambia, Uganda, and Kenya^20–22^. Unfortunately, the dissemination methods through social organizations and pharmacies cannot avoid offline contact and fully protect privacy. These two approaches mainly target fixed, intervenable small populations. E-commerce platforms, on the other hand, hold great potential for expanding the scope of SST. Nevertheless, despite existing research on the acceptability of SST and evaluations of on-site testing results among MSM and FSW, this is the first assessment of the diagnostic performance and service quality of online SST kits.

In order to quantify the service quality and credibility of the online SST kits, so as to serve as a reference for testers to choose products, we developed an independent score scale for online SST kits evaluation according to HONcode^16^. While the overall quality assessment scores indicated satisfactory performance across all test kits, several elements require optimization. Firstly, the testing tool should be integrated with the sampling tool to avoid the loss of sampling tool. Secondly, the self-sampling instructions and instructional videos should be provided by e-shop to ensure the correct self-sampling by using blood needles. Thirdly, the following explanations of the test results should be added to the instruction manual: (i) Considering that the window period for detecting syphilis through rapid testing is approximately 3 to 6 weeks, it is necessary to conduct at least a three-month follow-up after engaging in high-risk sexulal behavior if with negative test result. (ii) A positive test result could also indicate a previous infection. In such cases, it is necessary to seek further testing at an appropriate medical facility. (iii) Due to improper storage of reagents, improper operation, or being in the early stage of infection, faint positive bands may appear, making it difficult for patients to interpret the test results. In such cases, new reagents can be used to repeat the test, or patients can go directly to the hospital for testing. Forth, It is recommended that e-commerce platforms cooperate with medical institutions to provide end-to-end medical service, including sexual health education and consultation, SST kits sales, test results consultation, guide those who test positive to get further medical services and remind them to notify their sexual partners if they are diagnosed with syphilis.

Since 2010, Chinese policy has strengthened the combination of syphilis and HIV screening in all medical institutions and different populations. Nevertheless, the combination of SSTs with HIV self-tests remains uncommon. In this study, the number of after-sale comments for SST kits accounted for only 5.5% of those for HIV kits and 17% of those for dual-test kits for HIV and syphilis. Additionally, prior research has indicated that only 40% of MSM who self-test for HIV also self-test for syphilis. Conversely, almost all people who take self-tests for syphilis are also taking self-tests for HIV^14,18,23^, suggesting that the SST needs to integrate with existing HIV self-testing programs. In addition, after years of application and improvement of HIV self-testing kits, noninvasive self-sampling and rapid testing of blood, sweat, oral mucosal transudate, saliva, and urine can be achieved^24,25^. At present, there are studies out there that have evaluated *Tp* proteins in saliva or urine. Though, the performance has room for improvement^26,27^, respectively. Unfortunately, there are no studies on the detection of *T.pallidum* antibodies based on saliva and urine samples, so the development of non-invasive SST kits is still a great challenge. This will limit the wide application of SST.

Compared the diagnostic performance between eight online and nine facility-based syphilis testing, we found no difference in diagnostic performance between them other than ‘Abbott’. A recent meta-analysis showed the same results^28^. It’s worth noting that the sensitivity and specificity of all kits exceeded 90% and 95%, respectively, which reached the requirements of the WHO for the optimal detection performance of syphilis POCT^29^.

According to the requirements of WHO on POCT for syphilis^29^ and the updated “REASSURED” criteria^30^ for the ideal test that can be used at all levels of the health care system in the developing world to guide treatment and clinical management decisions for STIs, we proposed that SST should have characteristics named “ACCEPTIVE”, which will provide the basis for the health authority to judge whether this test method is suitable for the SST. “ACCEPTIVE” contains all the characteristics of screening tests, including ‘Cost-efficient’ (Cheaper than facility-based testing), ‘Valid interpretation with Rapid reaction’ (Results are available within 30 min and the interpretation is simple), ‘Ideally operate procedure’ (Simple testing procedure, no more than three steps. Don’t require any special device support.), ‘Effective in diagnostic performance’ (with Optimal Sensitive and Specificity over 90% and 95%, respectively). In addition, considering that the SST is self-sampled and operated, it should be sold on many e-commerce platforms or offline approach to improve the availability, as well as the privacy protection of testers and the correct clinical interpretation and medical guidance after self-testing are essential. The details were shown in Table 3.

**Table 3:** Characteristics of a ‘ACCEPTIVE’ diagnostic test.

Criteria	Description
Available	Available on many e-commerce platforms, health websites, etc.
Cost-efficient	Cheaper than facility-based testing.
Certified	Approved by NMPA.
Effective in diagnostic performance	Sensitive: Minimal >80%, Optimal >90%
	Specificity: Minimal >90%, Optimal >95%
Privacy protection	Privacy protected in packaging and delivery.
Tolerance of environment and sampling safety	Tolerate exposures between 2 °C and 45 °C at an altitude up to 3000 m, up to and including condensing humidity. Simple sampling procedure, provide sampling instructions and instructional videos. The non-invasive sampling is needed in the future.
Ideal operate procedure	Simple testing procedure, no more than three steps. Don’t require any special device support.
Valid interpretation with Rapid reaction	Results are available within 30 min, the results remain valid for at least 15 min, and the interpretation is simple.
Essential medical guidance	Correct clinical interpretation and medical guidance of test results are essential.

NMPA, national medical products administration.

There are several limitations in this study. Firstly, although over 30 distinct SST brands are commercially available on JD.com, our validation study assessed only eight of them. This limited sample size risks selection bias, necessitating expanded evaluation across the full product spectrum. Secondly, the diagnostic performance of the online kits in this study was validated in the laboratory, not in the field population. The results of the self-testing used by each tester may be affected by a variety of factors in the absence of unified training and guidance, which may cause different results between self-testing and facility testing. Therefore, further evaluation of the diagnostic performance of SST should be performed based on self-testing among target populations. Third, we did not assess the diagnostic performance of the kits for patients with syphilis at different stages, which need improve in future studies. Forth, the scientific and universal nature of the HONcode-based self-test service quality scale requires further validation across diverse disease conditions.

The good diagnostic performance and service quality of SST indicate that it can be used as an effective auxiliary test to expand the accessibility of syphilis testing services in key populations. However, there are still many problems with the application of SST, there is an urgent need to work with governments and health authorities to increase the credibility of testing, strengthen the publicity and improve its popularity and coverage, and to strengthen complementary sexual health counselling services, referrals and treatment services for those who test positive. It is necessary to further develop non-invasive self-sampling methods to improve the feasibility of self-testing. It is hoped that SST will play a greater role in the field of syphilis prevention and control in the future.

## Methods

### Search strategy

Considering that the manufacturers selling kits on different e-commerce platforms are the same group, we only searched for the sales of SST kits on JD.com, one of the largest e-commerce platforms in China, on the Google website until July 2025. We selected the top eight brands, named Accu News, Wondfo, WELLDAY, JISSBON, AIJI, KANGHUA, Abbott, and LOVCAE, in terms of sales volume and number of after-sales evaluations.

### Service quality assessment

Due to the lack of a global unified scoring system for the reliability and credibility of self-testing kits, we developed a score scale for the service quality of SST kits according to HONcode, a tool most widely used for determining the quality of health information on the internet^16^. This score scale consists of four sections, including Pre-test, Testing, Usability, and Follow-up. The ‘Pre-test’ section mainly evaluated brand popularity, price, delivery, and packaging. The ‘Testing’ section mainly evaluated whether the kit was approved by the National Medical Products Administration (NMPA), as well as recommended by the Chinese National guidelines^31^. The contents of the product instruction manual were evaluated in detail in the ‘Usability’ section. The ‘Follow-up’ section focuses on the interpretation of results, online consultation, and after-sales service. The max score was 50, and the service quality is divided into four levels: poor (< 25), average (25–30), good (30.5–40), and great (> 40).

### Information of after-sales evaluation

Two researchers searched for “JD.com” on the Google website and collected the after-sales evaluation information of these eight brands of online SST kits independently. The number of after-sales evaluations for each brand of kits has been counted and displayed by the platform. Comments were categorized as either positive or negative. Given the limited number of negative reviews, we focused on collecting and thematically classifying each negative review per brand.

### Study samples

The samples were randomly selected from the remaining inventory samples of previous project^32^, which was reviewed and approved by the Medical Ethics Committee of the Hospital of Dermatology, Chinese Academy of Medical Sciences (2019-KY-020). We confirmed that all methods were conducted in accordance with relevant guidelines and that informed consent was obtained from all participants. The remaining serum volume of each inventory sample was sufficient to meet the simultaneous testing requirements of all testing kits. As many samples as possible were included within the scope of the laboratory’s manageable workload. Initially, 330 samples were selected, but some samples did not meet the testing requirements, so the results of 319 (136 samples with treponemal antibody positive test by TPPA, and 183 samples with negative results) samples are presented.

### Diagnostic performance evaluation

All the samples were tested by using TPPA (Fujirebio, Japan), eight self-testing kits and nine facility-based testing kits according to the manufacturer’s instructions. The results of the TPPA test are used as the gold standard to evaluate the diagnostic value of these 17 rapid testing kits.

### Statistic analysis

Descriptive statistics were employed to characterize the information of online SST kits. The diagnostic performance evaluation of syphilis testing kits includes calculating their sensitivity, specificity, positive predictive value (PPV), negative predictive value (NPV), total coincidence rate (TCR), and Cohen’s Kappa coefficient, as well as their 95% Confidence interval (CI). The Cohen’s kappa coefficient value exceeding 0.8 indicates a near-perfect agreement^33^. The chi-squared test and Fisher’s exact test were used to compare categorical variables. A *P*-value of < 0.05 was considered statistically significant. All statistical analyses were conducted in SPSS 21.0 (IBM Corp, Armonk, NY).

## Supplementary Information

Below is the link to the electronic supplementary material.

Supplementary Material 1

## Data Availability

The sales and service information regarding the online SST kits in this study was obtained from the JD.com e-commerce platform (https://www.jd.com). The diagnostic performance test data for the eight online kits and the nine facility-based kits can be obtained from the corresponding author under reasonable requirements.

## References

[1] World Health Organization. Global progress report on HIV, viral hepatitis and sexually transmitted infections. (2021). Available at https://www.who.int/publications/i/item/9789240027077. Cited 8 September, 2025.

[2] Zhu, W. Q. et al. Analysis of the correlation between expanded syphilis screening and changes in epidemic reports in China from 2013 to 2021. *Chin. J. AIDS STD*. **28** (10), 1178–1182. 10.13419/j.cnki.aids.2022.10.14 (2022).

[3] Zheng, Y. et al. Syphilis epidemic among men who have sex with men: A global systematic review and meta-analysis of prevalence, incidence, and associated factors. *J. Glob Health*. **14**, 04004. 10.7189/jogh.14.04004 (2024). 38236688 10.7189/jogh.14.04004 PMC10795860

[4] Su, S. et al. Sustained high prevalence of viral hepatitis and sexually transmissible infections among female sex workers in China: a systematic review and meta-analysis. *BMC Infect. Dis.* **16**, 2. 10.1186/s12879-015-1322-0 (2016). 26732281 10.1186/s12879-015-1322-0 PMC4702370

[5] Du, F. Z., Zheng, Z. J. & Wang, Q. Q. Screening and referral for syphilis in non-STD clinics. *Chin. J. AIDS STD*. **25** (3), 320–326. 10.13419/j.cnki.aids.2019.03.29 (2019).

[6] Song, Y. et al. HIV-testing behavior among young migrant men who have sex with men (MSM) in Beijing, China. *AIDS Care*. **23**, 179–186. 10.1080/09540121.2010.487088 (2011). 21259130 10.1080/09540121.2010.487088 PMC3076143

[7] Krause, J., Subklew-Sehume, F., Kenyon, C. & Colebunders, R. Acceptability of HIV self-testing: a systematic literature review. *BMC Public. Health*. **13**, 735. 10.1186/1471-2458-13-735 (2013). 23924387 10.1186/1471-2458-13-735 PMC3750621

[8] Myers, J. E., El-Sadr, W. M., Zerbe, A. & Branson, B. M. Rapid HIV self-testing: long in coming but opportunities beckon. *AIDS* **27**, 1687–1695. 10.1097/QAD.0b013e32835fd7a0 (2013). 23807269 10.1097/QAD.0b013e32835fd7a0

[9] Zhang, T. P. et al. Community engagement in sexual health and uptake of HIV testing and syphilis testing among MSM in China: a cross-sectional online survey. *J. Int. AIDS Soc.* **20**, 21372. 10.7448/IAS.20.01.21372 (2017). 28406270 10.7448/IAS.20.01/21372 PMC5515028

[10] Ong, J. J., Fu, H., Smith, M. K. & Tucker, J. D. Expanding syphilis testing: a scoping review of syphilis testing interventions among key populations. *Expert Rev. Anti Infect. Ther.* **16**, 423–432. 10.1080/14787210.2018.1463846 (2018). 29633888 10.1080/14787210.2018.1463846 PMC6046060

[11] Steehler, K. & Siegler, A. J. Bringing HIV self-testing to scale in the United States: A review of challenges, potential solutions, and future opportunities. *J. Clin. Microbiol.* **57**, e00257–e00219. 10.1128/JCM.00257-19 (2019). 31462549 10.1128/JCM.00257-19 PMC6813024

[12] McGuire, M. et al. HIV self-testing with digital supports as the new paradigm: A systematic review of global evidence (2010–2021). *EClinicalMedicine* **39**, 101059. 10.1016/j.eclinm.2021.101059 (2021). 34430835 10.1016/j.eclinm.2021.101059 PMC8367787

[13] World Health Organization. WHO recommends HIV self-testing: evidence update and considerations for success. (2019). Available at https://www.who.int/publications/i/item/WHO-CDS-HIV-19.36 Cited 8 September, 2025.

[14] Wang, C. et al. Syphilis Self-testing: A Nationwide Pragmatic Study Among Men Who Have Sex With Men in China. *Clin. Infect. Dis.* **70** (10), 2178–2186. 10.1093/cid/ciz603 (2020). 31260513 10.1093/cid/ciz603 PMC7201417

[15] Wang, C., Li, X., Wang, Y. & Yang, B. Syphilis Self-Testing Among Female Sex Workers in China: Implications for Expanding Syphilis Screening. *Front. Public. Health*. **10**, 744240. 10.3389/fpubh.2022.744240 (2022). 35493357 10.3389/fpubh.2022.744240 PMC9045586

[16] Cardwell, E. T. et al. Web-Based STI/HIV Testing Services Available for Access in Australia: Systematic Search and Analysis. *J. Med. Internet Res.* **25**, e45695. 10.2196/45695 (2023). 37738083 10.2196/45695 PMC10559186

[17] Liu, F. et al. Availability and quality of online HIV self-test kits in China and the United States [abstract]. Presented at: Conference on Retroviruses and Opportunistic Infections; Feb 23–26; Seattle, United States. (2015). Available at https://www.croiconference.org/abstract/example Cited 8 September, 2025.

[18] Bil, J. P. et al. Usage of purchased self-tests for HIV and sexually transmitted infections in Amsterdam, the Netherlands: results of population based and serial cross-sectional studies among the general population and sexual risk groups. *BMJ Open.* **7**, e016609. 10.1136/sextrans-2018-053583 (2017). 28939577 10.1136/bmjopen-2017-016609 PMC5623511

[19] Ryan, A. et al. Range of self-tests available to buy in the United Kingdom: an Internet survey. *J. Public. Health*. **28**, 370–374. 10.1093/pubmed (2006). 10.1093/pubmed/fdl051 17052990

[20] Chanda, M. M. et al. HIV self-testing among female sex workers in Zambia: a cluster randomized controlled trial. *PLoS Med.* **14**, e1002442. 10.1371/journal.pmed.1002442 (2017). 29161260 10.1371/journal.pmed.1002442 PMC5697803

[21] Ortblad, K. et al. Direct provision versus facility collection of HIV self-tests among female sex workers in Uganda: a cluster randomized controlled health systems trial. *PLoS Med.* **14**, e1002458. 10.1371/journal.pmed.1002458 (2017). 29182634 10.1371/journal.pmed.1002458 PMC5705079

[22] Thirumurthy, H. et al. Promoting male partner HIV testing and safer sexual decision making through secondary distribution of self-tests by HIV-negative female sex workers and women receiving antenatal and post-partum care in Kenya: a cohort study. *Lancet HIV*. **3**, e266–e274. 10.1016/S2352-3018(16)00041-2 (2016). 27240789 10.1016/S2352-3018(16)00041-2 PMC5488644

[23] Zhong, F. et al. Acceptability and feasibility of a social entrepreneurship testing model to promote HIV self-testing and linkage to care among men who have sex with men. *HIV Med.* **18**, 376–382. 10.1111/hiv.12437 (2017). 27601301 10.1111/hiv.12437 PMC5340630

[24] Bacon, A. et al. Review of HIV Self Testing Technologies and Promising Approaches for the Next Generation. *Biosens. (Basel)*. **13** (2), 298. 10.3390/bios13020298 (2023). 10.3390/bios13020298 PMC9954708 36832064

[25] O’Byrne, P. HIV self-testing: A review and analysis to guide HIV prevention policy. *Public. Health Nurs.* **38** (5), 885–891. 10.1111/phn.12917 (2021). 34043831 10.1111/phn.12917

[26] Wang, C. et al. Quantified Detection of Treponema pallidum DNA by PCR Assays in Urine and Plasma of Syphilis Patients. *Microbiol. Spectr.* **10** (2), e0177221. 10.1128/spectrum.01772-21 (2022). 35315702 10.1128/spectrum.01772-21 PMC9045283

[27] Wang, C. et al. A New Specimen for Syphilis Diagnosis: Evidence by High Loads of Treponema pallidum DNA in Saliva. *Clin. Infect. Dis.* **73** (9), e3250–e3258. 10.1093/cid/ciaa1613 (2021). 33099614 10.1093/cid/ciaa1613 PMC8563222

[28] Towns, J. M. et al. The role of syphilis self-testing as an additional syphilis testing approach in key populations: a systematic review and meta-analysis. *Lancet Public. Health*. **8** (9), e726–e734. 10.1016/S2468-2667(23)00128-7 (2023). 37482070 10.1016/S2468-2667(23)00128-7

[29] World Health Organization. Point-of-care tests for sexually transmitted infections: target product profiles. Available at https://www.who.int/publications/i/item/9789240076234. Cited 8 September, 2025.

[30] Land, K. J. et al. REASSURED diagnostics to inform disease control strategies, strengthen health systems and improve patient outcomes. *Nat. Microbiol.* **4** (1), 46–54. 10.1038/s41564-018-0295-3 (2019). 30546093 10.1038/s41564-018-0295-3 PMC7097043

[31] National Center for STD Control, Chinese Center for Disease Control and Prevention & Group, V. Chinese Society of Dermatology, Subcommittee on Venereology, China Dermatologist Association. Guidelines for the diagnosis and treatment of syphilis, gonorrhea and chlamydia trachomatis infection. *Chin. J. Dermatol.* **53**, 168–179. 10.35541/cjd.20190808 (2020).

[32] Zhang, R. L. et al. The performance of a novel diagnostic criteria for neurosyphilis in HIV-negative patients. *Sci. Rep.* **14** (1), 31171. 10.1038/s41598-024-82477-6 (2024). 39732770 10.1038/s41598-024-82477-6 PMC11682042

[33] Li, M. & Yu, T. Methodological issues on evaluating agreement between two detection methods by Cohen’s kappa analysis. *Parasit. Vectors*. **15** (1), 270. 10.1186/s13071-022-05402-8 (2022). 35906628 10.1186/s13071-022-05402-8 PMC9338630

