# Supplementary material for: Evaluation of the service quality and diagnostic performance of syphilis self-test kits purchased from E-commerce platforms in China
Source: Sci Rep. 2026 Mar 22;16:14523. doi: 10.1038/s41598-026-44567-5 (PMC13149501; doi:10.1038/s41598-026-44567-5)
Supplement: Supplementary file 1 — Supplementary Material 1 [file 41598_2026_44567_MOESM1_ESM.docx]

**Supplementary Material**

I. Table S1. Information of eight online SST kits 2

II. Table S2. After-sales comments of 8 online syphilis self-testing kits 6

III. Table S3. Comparison of the diagnostic performance between online and faclility-based kits 8

**Table S1 . Information of eight online SST kits**

| **ID** | **Brand** | **Reagent name** | **Price (yuan/$)** | **Content** | **Requirements of sample** | **Test method** | **Storage** | **Expiration date** | **Interpretation of test results** | **No cross reaction mentioned** | **Product performance** | **Advantages** |
| --- | --- | --- | --- | --- | --- | --- | --- | --- | --- | --- | --- | --- |
| 1 | Accu News | Treponema pallidum Antibody Test Kit (Colloidal gold method) | 29/ 4.12 | Test card, buffer, disposable plastic straw, blood collection kit (Combined packing): disposable end blood collection needle, disposable disinfectant pad, band-aid | Avoid using hemolysis, hyperlipidemia, and hyperbilirubin samples. Cholesterol≤200mg/ml, Triglyceride≤200mg/ml, Hemoglobin≤170mg/ml, Bilirubin≤1.5mg/100ml | Fingertips, or venous blood Using fresh blood for test, or whole blood samples stored at 2-8℃ for no more than 48h | 2-30℃, keep dry and do not freeze | 24 months | Positive: C and T with red  Negative: C red, T no strip  Invalid: C No strip | Antibody of Hepatitis A, hepatitis B, and hepatitis C, rheumatoid factors | Negative reference conformity rate: ≥90% Positive reference conformity rate: ≥90% repeatability: repeated reference products were consistent for 10 times test Clinical positive coincidence rate: 99.18% Clinical negative coincidence rate: 100% Overall clinical compliance rate: 99.71% | Test card packaging with blood sampling tools. Had blood sampling instructions. |
| 2 | Wondfo | Treponema pallidum Antibody Test Kit (Colloidal gold method) | 30.25/4.30 | Test card, buffer, disposable plastic straw, blood collection kit (individual package): disposable end blood collection needle (Rotating cap), disposable disinfectant pad, band-aid | High concentration of jaundice samples (yellow in appearance), hemolysis of more than 25%, or chylous blood triglyceride content > 20.0mmol/L (white with flocculent deposits) will interfere with the test results | Fingertips, earlobe endings, or venous blood Using fresh blood for test. Or whole blood samples collected with an anticoagulant tube, and not suitable for testing if taken more than 7 days | 4-30℃ | 24 months, Use the test card within 1 hour when out of the package | Positive: C and T with red  Negative: C red, T no strip  Invalid: C No strip | none |  | - |
| 3 | WELLDAY | Treponema pallidum Antibody Test Kit (Colloidal gold method) | 22.9/ 3.25 | Test card, buffer, disposable plastic straw, blood collection kit (individual package): disposable end blood collection needle, disposable disinfectant pad, band-aid | serum bilirubin content should not exceed 200umol/L, the hemoglobin concentration in the whole blood sample should not exceed 18mg/ml, and the triglyceride should not exceed 25mmol/L | whole blood Whole blood samples collected with an anticoagulant tube, and not suitable for testing if taken more than 7 days | 4-30℃, keep in dark place, and do not freeze | 24 months, Use the test card within half hour when out of the package | Positive: C and T with red  Negative: C red, T no strip  Invalid: C No strip | HIV, HAV, HBV, HCV, HEV, EB, SLE, CMV, ANA, HTLV I/II, RF, influenza a virus | Negative reference conformity rate: 20/20 Positive reference conformity rate: 10/10 | - |
| 4 | Abbott | Treponema pallidum Antibody Test Kit (Colloidal gold method) | 37.05/5.26 | Test card, buffer, disposable plastic straw, blood collection kit (individual package): Painless blood needle, manual blood needle, disposable disinfectant pad, Band-aid | cholesterol≤245mg/dL, Bilirubin≤1.4mg/dL, RF(rheumatoid factors)≤28 IU/ml | Fingertips, or venous blood Squeeze the fingertip, insert with a sterile blood needle, wipe off the first drop of blood, then dip the open end of a new 20ul capillary straw into the next drop of blood, draw the blood into the capillary straw until to the black line, and drop into the test card | 2-30℃ | 24 months | Positive: C and T with red  Negative: C red, T no strip  Invalid: C No strip | Anti-Hbs, EBV, HIV, HAV, HTLV, HBsAg, CMV, HCV, borrelia burgdorferi, toxoplasma gondii, chlamydia, influenza, Schizotrypanum cruzi I/II, Schizotrypanum cruzi, Castellanella gambiense | Diagnostic sensitivity: 90.0% Clinical sensitivity: 99.67% Diagnostic specificity: 99.93% Clinical specificity: 99.72% | Had blood sampling instructions. Providing online consulting from clinicians. |
| 5 | JISSBON | Treponema pallidum antibody Test reagent (latex method) | 19.9/ 2.83 | Test card, buffer, disposable plastic straw, blood collection kit (individual package): disposable end blood collection needle, disposable disinfectant pad, band-aid | ascorbic acid≤ 20mg/dL, oxalic acid≤ 1000mg/dL, hematin≤ 1000mg/dL, Human Serum Albumin≤ 2000mg/dL, Triglyceride≤55mg/mL, Bilirubin≤60mg/dL | Fingertips, earlobe endings, or venous blood Using fresh blood for test, or whole blood samples using within 24 hours to avoid hemolysis | 4-30℃, keep in dark place, dry and do not freeze | 24 months, Use the test card within 1 hour when out of the package | Positive: C and T with red  Negative: C red, T no strip  Invalid: C No strip | HIV, HAV, HBV, HCV, HEV, RF, HAMA, ALT, HSV, prostate-specific antigen (PSA), H. pylori Ab, carcino-embryonic antigen (CEA), mononucleosis, toxoplasma gondii, CMV | Negative reference conformity rate: 100% Positive reference conformity rate: 100% Precision: The results of 10 parallel tests with precision reference materials were all positive with uniform chromaticity | low-cost |
| 6 | LOVCAE | Treponema pallidum Antibody Test Kit (Colloidal gold method) | 17.32/2.46 | Test card, disposable plastic straw, blood collection kit (individual package): disposable end blood collection needle, disposable disinfectant pad, band-aid | Triglyceride≤5.65mmol/L, Bilirubin≤1000umol/L, Hemoglobin≤6.5g/L | whole blood Using fresh blood for test | 4-30℃, keep in dark place, dry and do not freeze | 24 months, Use the test card within 1 hour when out of the package | Positive: C and T with red  Negative: C red, T no strip  Invalid: C No strip | antinuclear antibodies (ANA), RF, anti-mitochondrial antibody (AMA), HIV, HAV, HBV, HCV, HEV, HGV, toxoplasma gondii, rubella virus, EB, HSV I/II, CMV, influenza a virus | Negative reference conformity rate: 10/10 Positive reference conformity rate: 20/20 repeatability: repeated reference products were consistent for 10 times test Clinical positive coincidence rate: 100% Clinical negative coincidence rate: 99.70% Overall clinical compliance rate: 99.81% | low-cost |
| 7 | AIJI | Treponema pallidum Antibody Test Kit (Colloidal gold method) | 24.78/3.52 | Test card, buffer, disposable plastic straw, blood collection kit (Combined packing): disposable end blood collection needle, disposable disinfectant pad, band-aid | Triglyceride≤8mmol/L, Bilirubin≤300umol/L, Hemoglobin≤5g/L | Fingertips, earlobe endings, or venous blood Using fresh blood for test | 4-30℃, keep in dark place | 24 months, Use the test card within 1 hour when out of the package | Positive: C and T with red  Negative: C red, T no strip  Invalid: C No strip | HIV, HBV, HCV, RF | Negative reference conformity rate: 20/20 Positive reference conformity rate: 10/10 Precision: The results of 10 parallel tests with precision reference materials were all positive with uniform chromaticity | Test card packaging with blood sampling tools. |
| 8 | KANGHUA | Treponema pallidum Antibody Test Kit (Colloidal gold method) | 16.47/2.34 | Test card, buffer, disposable plastic straw, blood collection kit (individual package): disposable end blood collection needle (Rotating cap), disposable disinfectant pad, band-aid | Triglyceride≤5.65mmol/L, Bilirubin≤1000umol/L, Hemoglobin≤6.5g/L | whole blood Using fresh blood for test | 4-30℃, keep in dark place, dry and do not freeze | 24 months, Use the test card within 1 hour when out of the package | Positive: C and T with red  Negative: C red, T no strip  Invalid: C No strip | RF, Anti-HBs, HGV, HAV, HCV, HEV, HIV, SLE, tuberculosis antibody (TB-Ab) | Negative reference conformity rate: 10/10 Positive reference conformity rate: 20/20 Precision: The results of 10 parallel tests with precision reference materials were all positive with uniform chromaticity | low-cost |

Note: HIV,Human Immunodeficiency Virus; HAV, Hepatitis A Virus; HBV, Hepatitis B Virus; HCV, Hepatitis C Virus; HEV, Hepatitis E Virus; EB, Epstein-Barr; SLE, Systemic lupus erythematosus; CMV, Cytomegalovirus; ANA, Anti-Nuclear Antibody; HTLV, [Human T-cell Lymphotropic Virus](https://www.baidu.com/s?rsv_dl=re_dqa_generate&sa=re_dqa_generate&wd=Human T-cell Lymphotropic Virus&rsv_pq=8ccbbccd003d3445&oq=HTLV%E5%85%A8%E7%A7%B0&rsv_t=fa3a/k/I6e3csXU7W9W59+LW3ueNA3l4b04NCRTjV1fyNIQeMFOAWIQ4y5B4mTlrimVzBDvX&tn=57134193_oem_dg&ie=utf-8" \t "https://www.baidu.com/_blank); RF, rheumatoid factors; HAMA, Human Anti-Mouse Antibody; ALT, Alanine Aminotransferase; HSV, Herpes Simplex Virus‌; PSA, prostate-specific antigen; CEA, carcino-embryonic antigen; ANA, antinuclear antibodies; AMA, anti-mitochondrial antibody; HGV, Hepatitis G Virus; TB-Ab, tuberculosis antibody.

**Table S2. After-sales comments of 8 online syphilis self-testing kits**

| **ID** | **Brand** | **Total No. of comments for all products** | **Positive comments** | **No. of comments of Syphilis test kit** | **Proportion of comments of syphilis test kits** | **Positive comments** | **No. of Percentage of poor comments** | **Content of poor comments** | **No. of comments of HIV test kits** | **Proportion of comments between syphilis test and HIV test kits** | **No. of comments of HIV & syphilis test kits** | **Proportion of comments between syphilis test and HIV & syphilis dual test kits** |
| --- | --- | --- | --- | --- | --- | --- | --- | --- | --- | --- | --- | --- |
| 1 | Accu News | over 1,000,000 | 99% | 9,600 | 0.96% | High cost performance; Good quality, Privacy packing and Fast delivery, Satisfactory service attitude, Accompanied the whole test, Trustworthy. | 81(0.84%) | 1. No contents: 32 (39.51%) 2. Disposable blood collection needle is difficult to use and does not work: 5 (6.17%) 3. Suspicious of test results: 12 (14.81%) 4. Inconsistent test results with that performance in hospital: 12 (14.81%) 5. Quality problems, invalid detection, damaged blood collection needle, etc.: 3 (3.70%) 6. Delivery damage: 5 (6.17%) 7. Test kit packaging transparent, not enough attention to privacy: 5 (6.17%) 8. Doubts about the safety of disposable blood collection needles: 3 (3.70%) 9. Customer service guidance is not clear: 2 (2.47%) 10. Others: 2 (2.47%) | 190,000 | 5.05% | 57,000 | 16.84% |
| 2 | Wondfo | over 500,000 | 98% | 3,242 | 0.65% | Privacy delivery, Simple operation, First-line brand, trustworthy. | 42(1.30%) | 1. No contents: 11 (26.19%) 2. Disposable blood collection needle is difficult to use and does not work: 8 (19.05%) 3. Suspicious of test results: 7 (16.67%) 4. Inconsistent test results with that performance in hospital: 3 (7.14%) 5. Quality problems, invalid detection, damaged blood collection needle, sample diluent is empty, etc: 8 (19.05%) 6. Test tool packaging transparent, not enough attention to privacy: 1 (2.38%) 7. Kit content does not agree with the description: 2 (4.76%) 8. High price: 1 (2.38%) 9. Other: 1 (2.38%) | 48,220 | 6.72% | 15,180 | 21.36% |
| 3 | WELLDAY | over 50,000 | 98% | 104 | 0.21% | Good service attitude, Fast delivery | 4(3.85%) | 1. No contents: 1 (25%) 2. Suspicious of test results: 1 (25%) 3. Quality problem. The sample diluent is empty: 1 (25%) 4. Other: 1 (25%) | 11,150 | 0.93% | 8,150 | 1.28% |
| 4 | Abbott | over 20,000 | 99% | 703 | 3.52% | International brand, trustworthy, Satisfactory service attitude | 3(0.43%) | No contents: 3 (100%) | 3,216 | 21.86% | none | none |
| 5 | JISSBON | over 20,000 | 97% | 9 | 0.05% | Simple operation, Painless blood collection, Satisfactory after-sales service attitude. | 1(11.11%) | Suspicious of test results: 1 (100%) | 111 | 8.11% | 101 | 8.91% |
| 6 | LOVCAE | over 10,000 | 98% | none | 0.00% | none | none | none | none | none | none | none |
| 7 | AIJI | over 10,000 | 99% | 91 | 0.91% | Painless blood collection, Easy to interpret the results, Fast delivery | 1(1.10%) | No contents: 1 (100%) | 1,413 | 6.44% | 206 | 44.17% |
| 8 | KANGHUA | over 10,000 | 97% | 310 | 3.10% | Cheap price, Fast delivery | 10(3.23%) | 1. No contents: 2 (20%) 2. Sampling and testing tools are separate deliveries: 8 (80%) | 1,734 | 17.88% | 211 | 146.92% |

**Table S3. Comparison of the diagnostic performance between online and faclility-based kits**

| Online Brands | sensitivity | Facility-based Brands | sensitivity | *P-value* |
| --- | --- | --- | --- | --- |
| Abbott-syphilis | 94.85% | Abbott-HIV/syphilis | 91.91% | 0.465 |
| Abbott-syphilis | 94.85% | Egens | 98.53% | 0.172 |
| Abbott-syphilis | 94.85% | hemtrue | 99.26% | 0.066 |
| Abbott-syphilis | 94.85% | InTec | 100.00% | 0.014 |
| WELLDAY | 97.04% | Abbott-HIV/syphilis | 91.91% | 0.108 |
| WELLDAY | 97.04% | Egens | 98.53% | 0.447 |
| WELLDAY | 97.04% | InTec | 100.00% | 0.060 |
| AIJI | 98.52% | Abbott-HIV/syphilis | 91.91% | 0.019 |
| AIJI | 98.52% | Egens | 98.53% | 1.000 |
| AIJI | 98.52% | InTec | 100.00% | 0.247 |
| Online Brands | specificity | Facility-based Brands | specificity | *P-value* |
| Accu News | 98.90% | WANTAI | 97.81% | 0.685 |
| Accu News | 98.90% | HIGHTOP | 98.91% | 1.000 |
| Accu News | 98.90% | Abbott-HIV/syphilis | 100.00% | 0.248 |
| AIJI | 99.45% | WANTAI | 97.81% | 0.372 |
| Abbott-syphilis | 100.00% | WANTAI | 97.81% | 0.123 |
| Abbott-syphilis | 100.00% | InTec | 98.91% | 0.499 |
| Online Brands | positive predictive value | Facility-based Brands | positive predictive value | *P-value* |
| Accu News | 98.54% | WANTAI | 97.14% | 0.684 |
| Accu News | 98.54% | HIGHTOP | 98.55% | 1.000 |
| Accu News | 98.54% | Abbott-HIV/syphilis | 100.00% | 0.499 |
| AIJI | 99.25% | WANTAI | 97.14% | 0.371 |
| Abbott-syphilis | 100.00% | WANTAI | 97.14% | 0.123 |
| Abbott-syphilis | 100.00% | InTec | 98.55% | 0.499 |
| Online Brands | negative predictive value | Facility-based Brands | negative predictive value | *P-value* |
| Abbott-syphilis | 96.32% | Abbott-HIV/syphilis | 94.33% | 0.470 |
| Abbott-syphilis | 96.32% | Egens | 98.92% | 0.175 |
| Abbott-syphilis | 96.32% | hemtrue | 99.45% | 0.068 |
| Abbott-syphilis | 96.32% | InTec | 100.00% | 0.015 |
| WELLDAY | 97.85% | Abbott-HIV/syphilis | 94.33% | 0.112 |
| WELLDAY | 97.85% | Egens | 98.92% | 0.685 |
| WELLDAY | 97.85% | InTec | 100.00% | 0.123 |
| AIJI | 98.91% | Abbott-HIV/syphilis | 94.33% | 0.021 |
| AIJI | 98.91% | Egens | 98.92% | 1.000 |
| AIJI | 98.52% | InTec | 100.00% | 0.499 |
| Online Brands | Total coincidence rate | Facility-based Brands | Total coincidence rate | *P-value* |
| Abbott-syphilis | 97.81% | Abbott-HIV/syphilis | 96.55% | 0.474 |
| Abbott-syphilis | 97.81% | WANTAI | 98.75% | 0.545 |
| Abbott-syphilis | 97.81% | ABON | 99.69% | 0.069 |
| WELLDAY | 98.74% | Abbott-HIV/syphilis | 96.55% | 0.114 |
| WELLDAY | 98.74% | WANTAI | 98.75% | 1.000 |
| WELLDAY | 98.74% | ABON | 99.69% | 0.216 |
| Wondfo | 99.69% | Abbott-HIV/syphilis | 96.55% | 0.006 |
| Wondfo | 99.69% | WANTAI | 98.75% | 0.373 |
| LOVCAE | 100.00% | Abbott-HIV/syphilis | 96.55% | 0.001 |
| LOVCAE | 100.00% | WANTAI | 98.75% | 0.124 |
| Accu News | 99.37% | Abbott-HIV/syphilis | 96.55% | 0.021 |
| AIJI | 99.05% | Abbott-HIV/syphilis | 96.55% | 0.055 |
